# A first vocal repertoire characterization of long-finned pilot whales (*Globicephala melas*) in the Mediterranean Sea: a machine learning approach

**DOI:** 10.1098/rsos.231973

**Published:** 2024-11-06

**Authors:** M. Poupard, P. Best, J. P. Morgan, G. Pavan, H. Glotin

**Affiliations:** ^1^ University Toulon, AMU, CNRS, LIS DYNI, Marseille, France; ^2^ Centre International d'IA en Acoustique Naturelle, Toulon, France; ^3^ Longitude181, Valence, France; ^4^ Department of Computer Science, Swansea University, Swansea, UK; ^5^ Department of Earth and Environment Sciences, Universita di Pavia, CIBRA Interdisciplinary Center for Bioacoustics, Lombardy, Italy

**Keywords:** long-finned pilot whale, vocal repertoire, calls, classification, clustering

## Abstract

The acoustic repertoires of long-finned pilot whales (*Globicephala melas*) in the Mediterranean Sea are poorly understood. This study aims to create a catalogue of calls, analyse acoustic parameters, and propose a classification tree for future research. An acoustic database was compiled using recordings from the Alboran Sea, Gulf of Lion and Ligurian Sea (Western Mediterranean Basin) between 2008 and 2022, totalling 640 calls. Using a deep neural network, the calls were clustered based on frequency contour similarities, leading to the identification of 40 distinct call types defining the local population’s vocal repertoire. These categories encompass pulsed calls with varied complexities, from simplistic to highly intricate structures comprising multiple elements and segments. This study marks the initial documentation of the vocal catalogue of long-finned pilot whales in the Mediterranean Sea. Subsequent research should delve deeper into this multifaceted communication system and explore its potential linkages with social structures.

## 1. Introduction

Marine mammals and cetaceans in particular are capable of vocal production learning [1] and the calls they produce are drawn from a set of discrete categories: the vocal repertoire. Vocalizations are thus emitted as distinct units, that are non-randomly distributed over time [2]. The temporal distinction of units is commonly based on silent gaps between vocalizations. Unit types (or categories) are then defined based on similarity in spectral properties, such as the frequency contour. Generally, these repertoires are shared by populations or groups of individuals. For instance, the study of Ford [3] showed that killer whales (*Orcinus orca*) in the northeast Pacific have a unique vocal repertoire, with certain groups sharing a number of calls and others having a completely different set of calls. Other studies highlighted the acoustic repertoires of cetaceans such as bottlenose dolphins (*Tursiops truncatus*) [4], beluga whales (*Delphinapterus leucas*) [5], common dolphins (*Delphinus delphis*) [6] or long-finned pilot whales (*Globicephala melas*) [7].

Long-finned pilot whales reach an average length of approximately 6 m (with males usually larger than females). They are highly social and organized in matriarchal groups of 20–90 individuals [8]. The groups are stable over time, with individuals growing to maturity in their native group, in which they usually remain for life [9].

For some populations in different parts of the world, their vocal repertoire has already been described. In northern Norway, Vester *et al*. [7] characterized a repertoire with 129 distinct call types and 25 subtypes. In Australia, Courts *et al*. [10] found 18 different classes of vocalizations. In Nova Scotia (Canada), Nemiroff *et al*. [11] showed a high variation in call structures but did not define a repertoire. In the Mediterranean Sea, knowledge of long-finned pilot whales’ vocal repertoire is relatively limited (to the best of our knowledge, their vocal repertoire has never been published). Studies, such as Gannier *et al*. [12], have described variations of vocalizations’ frequency contour across species in Mediterranean delphinids, but do not define repertoire categories.

The characterization of a possible repertoire can give meaningful insights into the ecology of a species. In fact, communication systems are potential indicators of social structures [3], and have been conceptually and empirically attributed to social complexity (social complexity hypothesis) [13]. Indeed, for some avian and primate species, there seems to be a correlation between the complexity of communication and social interactions [14–16]. This motivates the study of non-human vocal behaviours, as a proxy to learn about local populations, their groups and how they might interact [17].

This study employed a semi-automatic methodology to delineate various call types. The approach involved several steps: initially manually detecting vocalizations within the acoustic recordings, subsequently applying an auto-encoder to extract distinctive vocalization features from these detections, and finally, clustering these features with manual validation and correction to establish the classification of distinct call types. Through this framework, we present an analysis of the call types observed in long-finned pilot whales across diverse areas within the Western Mediterranean Basin. Additionally, we explore their spectral–temporal characteristics and hierarchical classification.

## 2. Material and methods

In bioacoustics, the analysis of animal acoustic communication often involves five main steps: (i) data collection, (ii) vocalization detection, (iii) unit identification, (iv) sequence transcription, and potentially (v) function identification. In this study, we focused on the first three. To obtain as many long-finned pilot whale vocalizations as possible, we compiled recordings from various sources and organizations. First, vocalizations were manually detected in the available recordings. Then, we adopted the method proposed by Best *et al*. [18] to uncover and identify the new repertoire within the dataset.

### 2.1. Study site and field data collection

For this study, we used five different databases, listed in table 1. The oldest database is from 2008 (*Pavia*), while the latest is from 2022 (*WW*).

**Table 1.** Summary of the recording characteristics for each dataset. SR, sampling rate.

	data source				
	Pavia U	Sphyrna	L181	DYNI	WW
location (latitude, longitude)	Alboran Sea Italian coast	42°32’73 N, 4°21’18 E	42°35’8 N, 6°01’30 E	42°50 N, 6°20 E	43°4’43 N, 6°32’57 E
start–end time	2008-05-29 2009-08-07	2019-12-07	2022-06-23	2014-10-31	2022-06-23
recording system	Benthos AQ4 MOTU Traveler	Cet. Res. C75 HighBlue	Cet. Res. C75 HighBlue	GoPro H5	Cet. Res. C75 HighBlue
number of channels	2	5	1	1	4
SR (kHz)	192	256	22	48	256
depth (m)	18–20	4	10	2	10
recording time (h)	2:11	3:10	0:12	0:50	0:20
frequency range (kHz)	0.001 to 10	0.003 to 250	0.003 to 250	no	0.003 to 250
detection count	656	214	57	45	20

The first database (*Pavia*) was recorded using a custom-built towed array with two wideband preamplified hydrophones, connected by a detachable 240 m long cable to a PC workstation. The two hydrophones were 8 m apart and had a flat frequency response up to 40 kHz, extending to 80 kHz with a few dB roll-off. The array was towed from the stern of the *NRV Alliance* oceanographic vessel at a speed of approximately 5 kts, yielding an array depth of 18–20 m. The desktop PC workstation, equipped with the SeaPro software [19], provided real‐time monitoring and continuous recording of the two channels at 192 kHz, 16 bit. Acoustic detections were classified by taxon in real time by an expert operator with a 1 min resolution (Spectrogram NFFT = 2048, hopsize = 512).

The second database is from the *SPHYRNA* field expedition [20], which is described in the study of Poupard *et al*. [21]. Five hydrophones (3 Cetacean Res. CR75 and 2 CR57) were mounted under the ‘*SPHYRNA*’ Autonomous Surface Vehicle (ASV), surveying in the Western Mediterranean Basin for several months. The Qualilife HighBlue (QHB) sound card was used for acoustic acquisition [22]. The *SPHYRNA* data used in this study were acquired on 7 December 2019 between 23.00 and 02.00 local time, in the Gulf of Lion (France). Only the recordings from that day were analysed because they were of very good quality, showing animals on the surface to confirm the present species.

The last three databases (*L181*, *WW*, *DYNI*) were acquired during various marine mammal acoustic surveys off the French coast. The *L181* and *WW* databases were recorded with the QHB sound card and the *DYNI* database with a GoPro camera. Chapuis *et al*. [23] discussed the implications of using GoPro cameras for marine bioacoustic studies (acoustics index), highlighting the high sound quality they offer. Further inspection of spectrograms from such devices allowed us to confirm a sufficient quality to extract long-finned pilot whale calls. Vocalization signal-to-noise ratio (SNR) allowed a clear distinction of fundamental frequency contours in spectrograms and thus recordings were considered of sufficient quality to be used in the following methods. Table 1 shows the recording characteristics for each dataset. Sampling rates vary between 22 kHz and 256 kHz, all with at least 16 bits per sample. The number of detected vocalizations (last row) varies from 20 (*WW*) to 656 (*Pavia U*).

To create each database, hydrophones or cameras were deployed from a research vessel, after experts on board had visually identified long-finned pilot whales while checking for other species in the area. To ensure the safety of the animal, an ethical approach was used by those onboard the research vessel, where

— a minimum distance of 100 m between the animals and the vessel was maintained (unless the whales came closer by their own volition),
— the vessel always approached the animals slowly and sideways, never from the front or rear, and
— a group of cetaceans were never split by the vessel.

**Figure 1.**
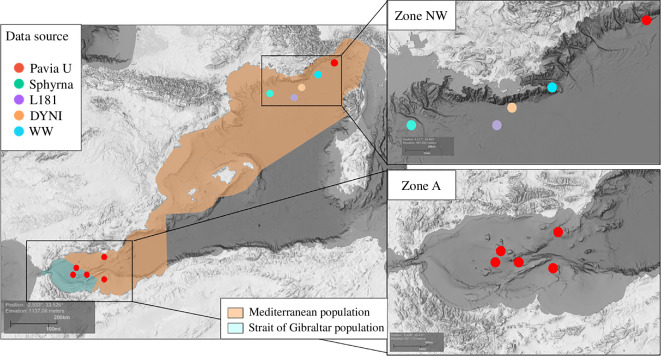
Map of the Mediterranean region and recorder locations. Each dot colour represents a database. The coloured areas are the two populations of long-finned pilot whales identified by Verborgh *et al*. [24] based on genetic structure and individual movements. Source of the bathymetry data [25,26].

The geographical position of these recordings is shown in figure 1. Two large areas were covered: the Alboran Sea (zone A) and the northwestern Mediterranean (zone NW). Only recordings from the Pavia database were made within zone A, indicated by the red dots on the map. This area presents specific oceanographic characteristics due to the transition between the Mediterranean Sea and the Atlantic Ocean. The northeastern Alboran Sea is an important feeding and breeding ground for some cetacean species (bottlenose dolphin *Tursiops truncatus*, common dolphin *Delphinus delphis* and long-finned pilot whales) [27,28]. All other recordings, including the remaining of Pavia’s, are located in the northwest Mediterranean Sea (from the Gulf of Lion to San Remo, Italy), along the continental slope (zone NW).

In the Mediterranean Sea, the approximate distribution of long-finned pilot whales was described in a study conducted by Verborgh *et al*. [24]. Specifically, they observed a higher density in the Alboran Sea (zone A in figure 1) as compared to the northwestern Mediterranean (zone NW in figure 1). It is difficult to confirm that encounters from zones A and NW relate to two different populations, but the study of Verborgh *et al*. [24] showed there are two genetically distinct populations of long-finned pilot whales in the Mediterranean Sea. The first, the Mediterranean population (orange on the map), extends from the east of Djibouti Bank and the Alboran Dorsal up to the Ligurian Sea, while the second, identified as the Strait of Gibraltar population (blue), remains in the eponym area. Thus, observations from zone NW can be attributed to the first population, but those from zone A could be of the two populations.

### 2.2. Sound analysis

To ensure a uniform analysis across databases, only signals below 10 kHz (below the Nyquist frequency for recordings at 22.05 kHz) were analysed (databases with varying sampling rates were used: 22.05 kHz to 256 kHz). All databases were small enough for vocalizations to be detected manually. To this end, each recording was analysed using Audacity software. A spectrogram was created (NFFT = 2048, hopsize = 512) to visualize the signal while listening to it. Once a vocalization was detected by the user, a time annotation was made and extracted in .txt format from Audacity. Two signals were considered distinct vocalizations if at least 0.5 s separate them.

**Figure 2.**
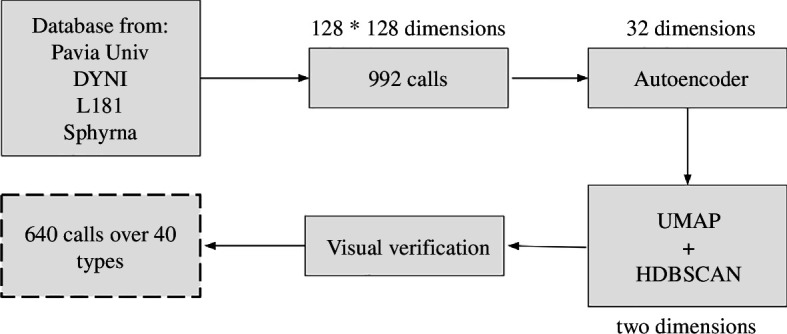
Schematic diagram of the method. The 992 vocalizations detected with all databases were analysed via the auto-encoder. Among these vocalizations, 640 were categorized in 40 types.

The different stages of signal analysis are shown in figure 2, the first one being the detection of long-finned pilot whale calls.

#### 2.2.1. Dimensionality reduction

After calls have been identified in the recordings, the next stage is the categorization into call types. To aid the categorization of calls and compromise between reliability and efficiency, a mixture of automatic procedures and manual verification were employed.

We first represent vocalizations by spectrograms (using windows of 43 ms and 80% overlap) and then reduce their dimensionality to better measure similarity. To do so, we follow the practice as shown by Best *et al*. [18], where two complementary reduction methods are employed in series.

Firstly, an auto-encoder was trained to compress vocalization spectrograms into 32 dimensions, while preserving enough information to be able to reconstruct them. The goal of an auto-encoder is to minimize the difference between the input data and the reconstructed data. Once the auto-encoder is trained, we can use the bottleneck embedding to represent vocalizations. Hence, after training, the encoder subnetwork was used to project vocalizations into 32 dimensions.

Secondly, the Uniform Manifold Approximation and Projection (UMAP) algorithm [29] allowed the dimensionality to be further reduced to 2 (avoiding the curse of dimensionality in distance measurements [30]). UMAP aims to preserve the underlying structure of the data by mapping it to a lower-dimensional space while maintaining the local and global relationships between data points (like principal component analysis [31] and t-SNE [32] reduction).

Using these two methods for dimensionality reduction has been shown to provide more optimal results than one alone [18]. The auto-encoder, while trained on a reconstruction loss, can reduce noise while preserving the structural information of the data. However, if the auto-encoder was used to compress the data to two dimensions directly, the bottleneck would be too extreme for an accurate reconstruction. Therefore, by first employing the auto-encoder and then applying UMAP to the result, we achieved a better overall representation in a lower-dimensional space.

This lower dimensional space not only allows the vocalizations to be visualized in a scatter plot (where each point represents a vocalization), but can also be used to measure their similarity. In this representation, vocalizations that are proximate to each other will exhibit similar spectro-temporal patterns and are likely to be associated with the same type. Such vocalization representation has shown good agreement with the expert categorization of calls from different species, including an odontocete [18]. Thus, distances in this lower-dimensional space are correlated with similarity of frequency contours, and can be used to help in repertoire characterization.

After UMAP was used to reduce the number of dimensions to 2, points were clustered using the HDBSCAN algorithm [33]. By suggesting a preliminary categorization with these clusters, we reduce the human effort needed to group similar vocalizations into call types. Clusters can then be manually corrected (merging clusters together or sorting them into subcategories) to produce the final categorization of all vocalizations.

The validation of clusters and attribution of vocalizations to call types was based on: (i) their morphological shape; the overall form of the vocalization was the first aspect considered via audio and visual inspection. (ii) Their acoustic features, such as the fundamental frequency (F0), segments, elements and duration [7,34,35]. This second step requires comparing their features directly on the spectrogram. (iii) Finally, the quality of the clusters was also assessed by a visual inspection of their dispersion (intra/inter-cluster variance) during the final validation of the clusters.

This method is supported by different studies that have shown the reliability and complementarity of aurally and visually identifying acoustic repertoire [35–37]. In the case of low SNR and/or overlapping vocalizations, the data made it so it was difficult to precisely identify the vocalization. So, some of them were excluded from the analysis. This was a qualitative assessment made by the ear of the human operator. Then, we kept only types of calls that were present at least three times in the analysis. So, ‘rare’ calls were also excluded. After excluding these samples, 640 out of the original 992 vocalizations were left for classification into call types.

#### 2.2.2. Pitch extraction

Once call categories were attributed, a custom interface^1^ was used to annotate their F0 contour (a common practice to compare tonal and harmonic calls [38,39]). Several studies have automatically extracted the vocalizations’ F0, but these methods are not robust to noise [40] and are not reliable enough for this database. Indeed, in low SNR and in the presence of overlapping clicks and vocalizations, the F0 estimations sometimes lose the targeted harmonic/vocalization [41].

**Figure 3.**
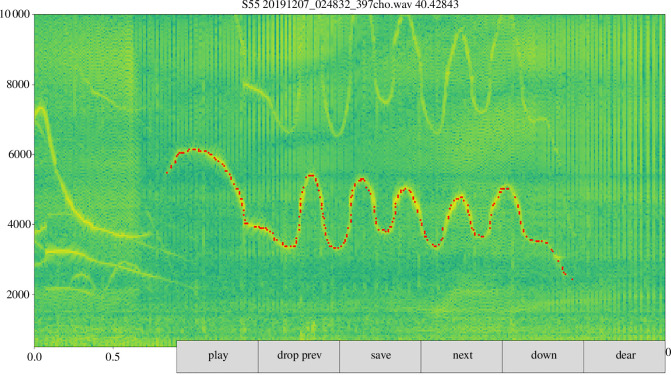
Demonstration of the F0 annotation tool. The red dots have been added by the annotator.

Figure 3 shows the custom interface, in which a user draws F0 contours on spectrograms with a computer mouse. As so, thre vocalizations per type were annotated. The operator can play the audio, delete their selection and switch harmonics (divide the frequency of all points by 2). The latter operation is useful when the first harmonic is more prominent than the fundamental. The operator can then outline the first harmonic and divide frequencies by 2 to place it back on the fundamental. In general, to identify the F0, we made sure that there was a harmonic at twice its frequency.

### 2.3. Statistical analysis

Once the F0 contours were extracted, they were used for statistical analysis of call characteristics across databases and for their hierarchical classification. To compare the different databases, we measured four acoustic parameters from the annotated F0 contours:

— Duration (time between onset and offset of the call).
— Maximum frequency.
— Minimum frequency.
— Mean frequency.

These four parameters were tested for significant differences between the datasets (the parameters of calls exclusive to one dataset were compared to those of others).

Additionally, previous studies of long-finned pilot whale vocalizations have found the mean, minimum, and maximum frequencies to be highly correlated [42]. We tested the correlations between these three parameters using Spearman’s correlation [43]:

— Correlation between the minimum and maximum frequencies.
— Correlation between the minimum and mean frequencies.
— Correlation between the mean and maximum frequencies.

The *WW* and *DYNI* databases were not statistically analysed because of their low number of samples (one type for each database). *L181* was also excluded from the analysis, as no call type belonged exclusively to this database.

The first test was the Shapiro–Wilk test to assess the normality of distributions. If acoustic parameters were normally distributed, a parametric test (ANOVA; analysis of variance) was used to compare their distributions across datasets; if not, a non-parametric test (Kruskall–Wallis) was used to assess the statistical significance of the distribution difference. If the *p* value of the Kruskall–Wallis test is ⩽α, it indicated the differences between some medians are statistically significant. A *post hoc* test was then used to assess the differences between each distribution (Bonferroni test).

### 2.4. Classification tree

Our study’s final analysis was the hierarchical classification of Mediterranean long-finned pilot whales’ vocal repertoire using a classification method called non-parametric classification tree (CART) analysis [44].^2^ Such classification trees have shown great promise for classifying the vocal repertoires of several marine mammal species: humpback whales [17] (*Megaptera novaeangliae*), beluga whales [5], dolphins [48] and bearded seals (*Erignathus barbatus*) [49].

CART is robust to outliers and is preferable to principal component analysis, which requires homogeneity of variances and linearity [17]. Applying CART yields a decision tree where each fork is split by a predictor variable and each node has a prediction for the target variable at the end. During the construction of the tree, all variables are considered in each split decision, here by using the Gini index [50].

For CART’s input variables, we used the same acoustic parameters as in the study of Garland *et al.* [5] on beluga whales. Some were inferred from F0 measurements, and some were measured manually from spectrogram visualization (numbers of inflections, segments and elements):

— Duration (s) (Dur; Length of call).
— Bandwidth (Hz) (BW; Min/Max frequency of the fundamental (F0)).
— Peak frequency (Hz) (Peak; Frequency of the spectral peak).
— Frequency range (ratio) (Range; Ratio of max/min frequency).
— Frequency trend (ratio) (Trend; Ratio of start/end frequency).
— Inflections (Inflec; Number of reversals in slope).
— Segments (Segm; Number of segments).
— Elements (El; Number of elements).

Segments are units within a vocalization separated by a short silent gap (less than 0.1 s), whereas elements are units within a segment separated by an abrupt frequency shift (without silent gap). The bandwidth was calculated by subtracting the minimum frequency from the maximum frequency of the fundamental, without taking into account the harmonics because they were sometimes not visible and/or cut off by the low sampling frequency.

We did not include the maximum, minimum, start and end frequencies in the CART analysis because they did not affect the classification (identical trees with and without these parameters). These parameters were also correlated with each other (see Pearson correlation in the results). Furthermore, it ensured calls were classified by F0 shape and not F0 position (in the frequency space).

## 3. Results

The results of this study are divided into three main sections: repertoire identification, repertoire analysis and hierarchical classification of the vocalizations. The repertoire identification involves clustering and manual sorting, the repertoire analysis was based on features derived from the F0 contours and their statistical comparison, and the hierarchical classification was based on a CART.

### 3.1. Repertoire identification

**Figure 4.**
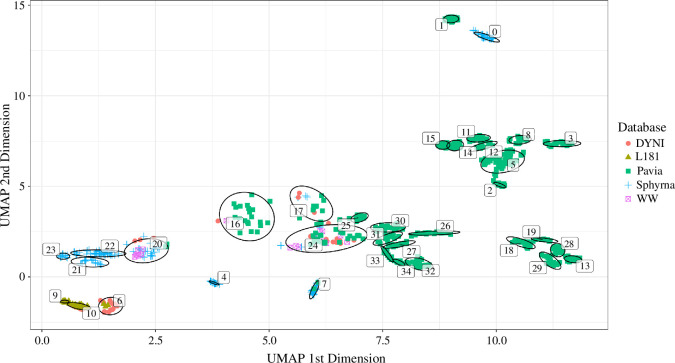
UMAP projection of auto-encoder embeddings, with each point being a vocalization’s projection. Different groups (ellipses with number identifiers) represent the different clusters found by the HDBSCAN using the two dimensions. Each symbol and colour represent a database. Non-clustered points are excluded from the visualization.

The auto-encoder and UMAP dimensionality reductions followed by HDBSCAN clustering yielded 35 distinct clusters (figure 4). All of them were inspected aurally and visually to correct them for potential outliers, to split or to merge them. Some 181 points were not found to belong to any cluster by the HDBSCAN algorithm, but were also analysed to see if they contained any new vocalization type. They mostly consisted of vocalizations with low SNR or overlapping vocalizations, making them too difficult to classify.

In the recordings, clicks and whistles (above 10 kHz) were observed, but only pulsed calls and whistles below 10 kHz were classified. Through the inspection of clusters, 40 different call types were distinguished, gathering 640 vocalizations (some vocalizations could not be classified because of either a too-low SNR or overlap with other vocalizations). This means that overall, 65% of the database was categorized. Note that call type labels were attributed according to the categorization process and have no semantic or hierarchical value. Spectrograms for each call type are shown in electronic supplementary material, figure A.

The call types encountered have a variety of shapes (ascending, descending or alternating between the two), with some containing harmonics and others not. Some calls also comprise several segments [7,42] and/or several elements.

**Figure 5.**
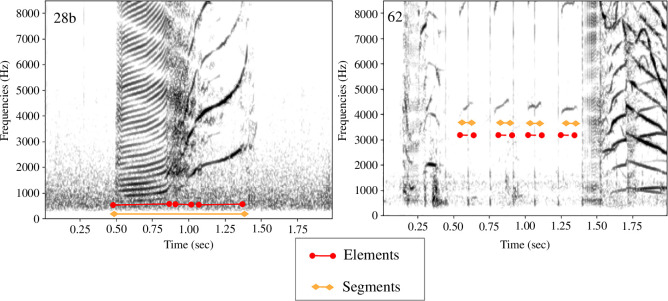
Two spectrogram examples of long-finned pilot whale types (28b and 62) with their elements and segments. For example: call 28b consists of three elements (constant, atonal and ascending) and one segment.

For instance, call type 28b consists of several elements (three) and only one segment, while type 62 consists of four elements (figure 5). Type 62 has been grouped into a single call type due to the segments being separated by a very short silence. This decision was also influenced by the study from Yurk [35], which defined types containing multiple identical segments (such as AKS21, produced by AB- and AD-clan).

**Figure 6.**
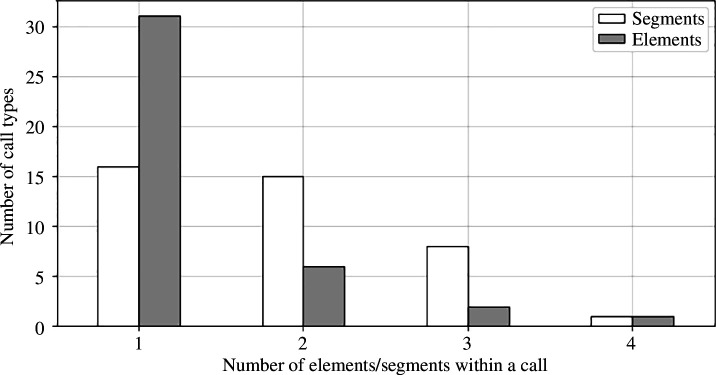
Number of elements/segments per call type of long-finned pilot whale.

The number of elements and segments of a given call type will reflect its complexity within the directory. Their distribution across all call types is shown in figure 6. The number of elements and segments ranges from 1 to 4, with an average of about 1.325 and of 1.85, respectively. The majority of types have one element (77%). Out of 40 types, 24 have more than 1 segment (60%), which shows the complexity of the repertoire in terms of number of segments. Conversely, only 9 of the types have more than 1 element (22%).

**Figure 7.**
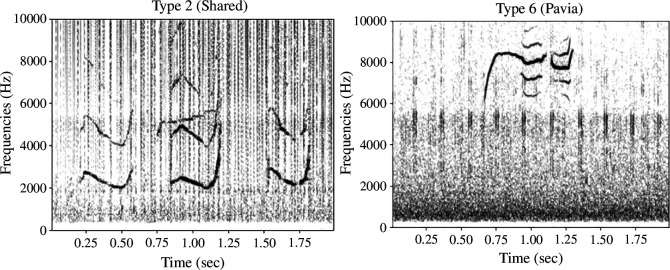
Spectrograms of the two most represented types of vocalizations (type 6 and type 2).

**Figure 8.**
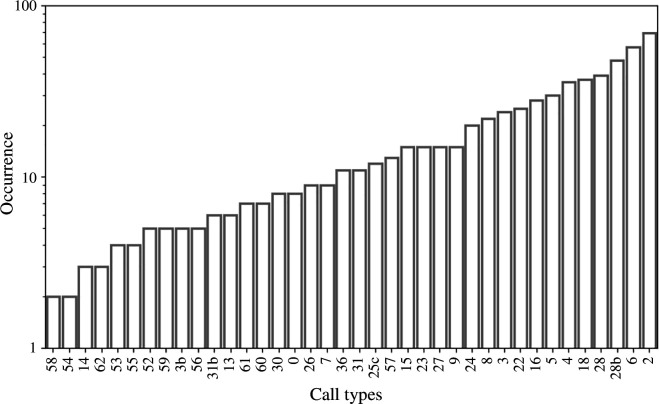
Occurrence of each detected type (logarithmic scale).

Figure 8 represents the number of occurrences of each type. Among the 40 defined types, some are poorly represented (types 58, 54), while others are more common, such as type 6 and type 2. Figure 7 shows the spectrograms of vocalization types 6 and 2. Type 6 was recorded only in the Pavia database, while type 2 was recorded in several databases (figure 8).

The average occurrence per call type is 18 vocalizations.

### 3.2. Repertoire analysis

Thanks to the manual F0 contour estimation, we were able to measure mean, minimum and maximum frequencies as well as the duration for each type of the repertoire. The mean frequency of the classified types ranges from 836.5 Hz (type 23) up to 7866.8 Hz (type 6) (see table 2). The call type duration is also reported in table 2, the shortest being type 60 (0.21 s), and the longest type 18 (1.36 s).

**Table 2.** Statistical measurements of call types.

	call type measure	
statistic	frequency (Hz)	duration (s)
minimum	836.5	0.21
maximum	7866.8	1.36
mean	3449.8	0.84
standard deviation	1358.1	0.24

**Figure 9.**
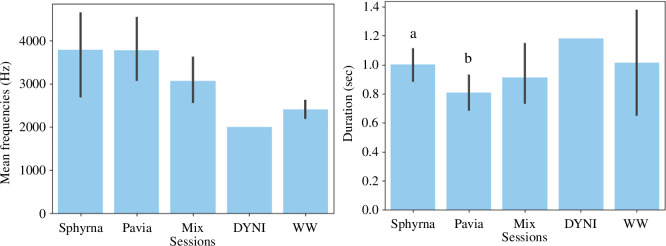
Bar plot of mean frequencies (left) and durations (right) across databases. The ‘Mix’ category denotes call types present in multiple databases.

Various statistical tests were performed to compare call types across databases. Call types that were exclusive to one database were labelled accordingly (Sphyrna *n* = 7 and Pavia *n* = 18), and those present in multiple databases were labelled as ‘mixed’ (*n* = 12). Neither WW, DYNI, nor L181 were included because of their low number of samples (they had only one or no exclusive call type). As the data were not normally distributed for the duration (*p* = 0.027 ≤α, H statistic = 0.975), a Kruskall–Wallis test was performed: durations were significantly different between databases (*Pavia* and *Sphyrna*) (*p* = 0.011 ≤α, H statistic = 14.882). Calls from the *Pavia* database are thus significantly shorter than those recorded on *Sphyrna* (figure 9).

As for mean frequencies, the normality of the distribution was confirmed (*p* = 0.272 ≥α, H statistic = 0.966), so an ANOVA test was performed: mean call frequencies were not statistically different between databases (*p* = 0.776 ≥α, H statistic = 0.471).

**Figure 10.**
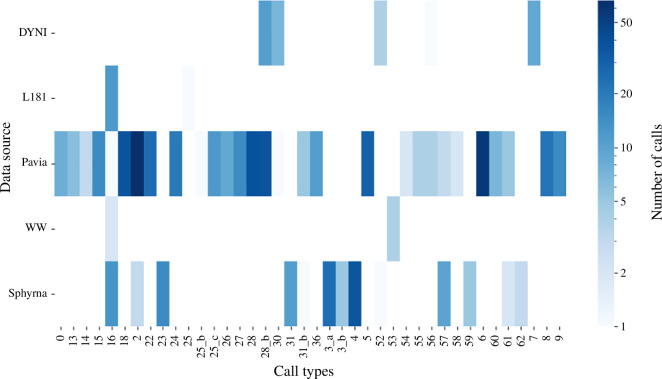
Heatmap of the classified types according to the data source. The colour-map is on logarithmic scale. *n* = 32 for DYNI; *n* = 13 for L181; *n* = 462 for Pavia; *n* = 6 for WW; *n* = 129 for Sphyrna.

Figure 10 presents the occurrence of call types across all databases. Most of the calls were detected in the Pavia database (66%), followed by Sphyrna (21%), DYNI, L181 and WW each making less than 10% of the total detected calls. Most call types are exclusive to one database, except 8 of them (20%): 16, 2, 28b, 31b, 52, 56, 57 and 61.

While we do observe differences within call types between north and south populations, more data are needed to make statistical analysis and assess the impact of geographical variations. However, it is worth noting that the zone A recordings were made from 2008 to 2009, while other recordings were made from 2014 to 2022, so the possibility of temporal variations cannot also be ruled out. More data would be needed to statistically test the temporal and geographic evolution of the repertoire.

### 3.3. Hierarchical classification

**Figure 11.**
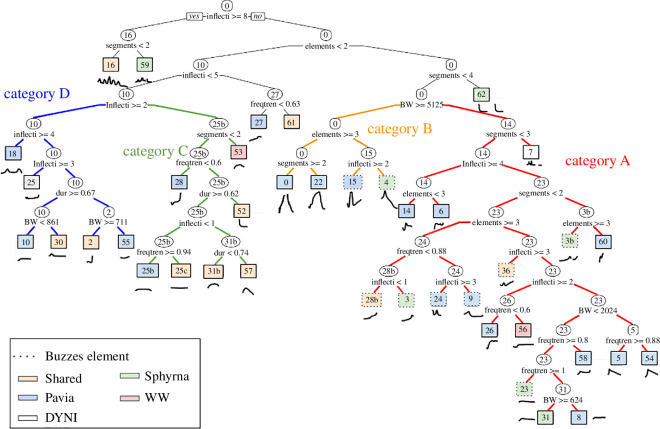
Classification and regression tree (CART) of Mediterranean long-finned pilot whale calls. The variables used at each tree division are indicated along with the criteria used (<, >). All lines at the left of the split fulfil its condition, and lines at the right do not. Variables' abbreviations can be found in §2.4. Each leaf colour represents a database, and dotted boxes indicate calls with buzzes. Types 59 and 16 do not belong to any category and stand apart in the tree because of their high complexity, consisting of more than eight inflection points. Global Gini Index = 0.37.

The final analysis of this study was the classification tree of the 40 call types (CART; figure 11). Such representation makes it possible to test whether a call from a new recording is part of this repertoire or not. The eight variables used in this tree are detailed in §2.4.

The first acoustic parameter that divides the vocalizations into two clusters is the number of inflection points (> 8). To the right of the tree (category A, B, C and D) are vocalizations with a number of inflection points that are lower than 8. The only two vocalizations with a number of inflection points greater than 8 are 16 and 59; they are highly complex and do not belong to any category.

The second parameter that separates categories C and D from categories A and B is the number of elements in the vocalization (< 2). Categories A and B are, therefore, composed of vocalizations with more than two elements and are relatively complex, particularly for category B with more than three elements.

## 4. Discussion

This study provides the first description of long-finned pilot whales’ vocal repertoire in the Mediterranean Sea. It makes use of innovative methods involving deep neural networks to unveil 40 different call types from 640 detected vocalizations, and reports them in a classification tree based on F0 contour features.

This work does not claim to be a definitive repertoire, but rather serves as an initial framework for future bioacoustic research on this species. Indeed, this catalogue cannot be complete or definitive as it does not cover the entire Mediterranean Sea and only includes signals below 10 kHz.

### 4.1. Comparison with other repertoires

The vocal repertoire of the long-finned pilot whale is a relatively rich one as compared to other species. In this study, we found 40 distinct types. As a comparison, in Norway, Vester *et al*. [7] identified 129 different distinct types and 25 subtypes; in Nova Scotia, Nemiroff *et al*. [11] showed a quantitative description of the pulsed call structure and highlighted their acoustic complexity; and in Australia, Courts *et al*. [10] found a smaller repertoire of 18 classes over 2028 calls. These studies have shown, as we have, that the acoustic repertoire of this species can be highly complex.

In the Bahamas, Sayigh *et al*. [51] have also described a vocal repertoire with the short-finned pilot whale (a close relative to the long-finned pilot whale). Out of 4098 calls, 173 types were defined. The large variability in repertoire size for a single species can result from many factors. Some affect the production of vocalizations and are listed in table 3. Others arise from the different methodologies employed by researchers and the criterion used in defining distinct categories. Furthermore, with a relatively small sample of a thousand vocalizations, our repertoire size might be underestimated here.

**Table 3.** List of factors that may influence the composition of a vocal repertoire in toothed cetaceans and associated bibliography.

species	influential parameters	bibliography
long-finned pilot whale	behaviours	[ 7,52]
	group composition	[11,52]
	geographical location	[53]
	human presence (noise)	[54,55]
	other species presence	[56]
	environment categories	[54]
bottlenose dolphin	seasonality/temporal change	[57,58]

Calls can vary not only in frequency but also in amplitude. Indeed, Miller [59] found the killer whale vocal repertoire could be divided into ‘long-range’ pulsed calls (10–16 km) and ‘short-range’ sounds (5–9 km) with different emission levels, and these two types of call correspond to certain behaviours. Short-range sounds were produced more during social and resting behaviours, whereas long-range stereotyped calls predominate in dispersed travel and foraging behaviours. Therefore, the dB level could be another parameter to be taken into account in the study of long-finned pilot whales’ vocal repertoire.

### 4.2. Repertoire and calls characteristics

The 40 calls in the repertoire appeared with varying frequencies. Types 2, 6, and 28b account for over 27% of the total number of calls, whereas vocalizations 58, 57 and 14 represent only 1% of the total calls. The frequent appearance of specific types can be explained in multiple ways. For type 2 and type 6, they were mostly observed in repetition (from 2 to 6 times), which explains their high abundance in recordings. In fact, the repetition of the same unit could influence the receiver’s reactions and could carry a different meaning. Often, repetitions are used as an alarm to warn of a predator or in the context of noise [15,60]. The study conducted by Zwamborn & Whitehead [52] investigated the relationship between surface behaviour and repetitive calls, and found that they serve to maintain contact and cohesion during social behaviour.

The analysis of the F0 contour for each call type allowed us to describe the repertoire based on acoustic features. The mean frequency of calls varies between 0.836 and 7.866 kHz, and the global mean is 3.449 kHz (table 2). In Norway, the average frequency of calls was 4.23 kHz (mean of high and low clusters) [7]. Average call frequencies are, therefore, relatively similar between Mediterranean and Norwegian pilot whales (590 Hz difference). But it is important to take into account that in Vester *et al*.’s [7] study, calls above 10 kHz were also considered. In the future, higher sampling rate will be taken into account in order to capture higher frequency patterns.

Concerning the minimum frequencies, in our study the average minimum was 2.3 kHz. There was a big difference between the populations of the Northern and Southern Hemispheres. Indeed, in the Northern Hemisphere, the minima range from 2.5 kHz to 3.5 kHz [7,61,62], while in Australian populations, the minimum frequency was almost 1 kHz higher (4.2 kHz) [10].

Our study, therefore, confirms the hypothesis that pilot whales in the Northern Hemisphere have a lower minimum call frequency (2.3 kHz) than those in the Southern Hemisphere (4.2 kHz, in the study of Courts *et al*. [10]). Differences in frequency ranges could be due to physiological, behavioural and/or environmental differences [62].

### 4.3. CART classification

Extracting pitch parameters for each call type also allowed us to build a hierarchical classification of Mediterranean long-finned pilot whale calls. This method was used for several cetacean species such as beluga whales [5], nine species of delphinids [48], humpback whales [17,63], as well as long-finned pilot whales [54]. Similar to our findings, previous studies also recognized the challenges in categorizing discrete types due to sometimes graded acoustic structure [64,65]. They demonstrated this classification could be used to comprehend the organization of different calls, as we performed in our study (figure 11). Based on the vocalizations’ shape, the CART highlighted four main categories (A to D) and enabled us to discriminate call types within these categories. Initial discriminating parameters included the number of elements, segments and inflection points, followed by frequency parameters. It should be noted that some calls are specific and do not belong to any category (e.g. type 16 and 59). These calls have very complex structures, such as inflection points greater than 8 or a number of elements greater than 4 and could have particular functions for communication.

Graded calls that lie between types could be observed in the future, which would question the discreteness of the repertoire. The classification presented here thus gives a first structure, which could be completed in the future, potentially with continuous variations around frequency contours. In fact, our method is based on a ‘discrete’ classification of calls, i.e. each vocalization is associated with a call type, but some recent studies have shown that many vocal repertoires exhibit graded morphology, suggesting that the acoustic structures of vocalizations are not clearly separated and discrete, but form a continuum in the acoustic space [66]. Nonetheless, the CART analysis provides a reliable classification method with an interpretable and visual output, which reduces potential annotator biases. The average Gini index across all nodes in the decision tree is 0.37, which reflects a fairly good level of class separation. While not perfect, this indicates that the model is already performing well in distinguishing between the different vocalizations. This classification could still be improved by including harmonics as an input parameter (they were shown to carry information in long-finned pilot whale communication [67]).

### 4.4. Parameters influencing repertoire

Vocal repertoires produced by cetaceans can be variable and more or less extensive [68–70]. The composition and size of these catalogues may depend on a number of parameters. Table 3 lists these main parameters with the corresponding bibliography on the long-finned pilot whale.

The behavioural state of the group is the first factor influencing the composition of the repertoire. The study by Vester *et al*. [7] demonstrated a link between the production of complex calls and surface behaviour in long-finned pilot whales. Cetaceans are likely to communicate specific information about their activity by producing certain types of calls. For instance, whistles and complex pulsed calls are associated with active surface behaviours such as body contact during multi-pod aggregations [7]. In the future, it will be essential to record the surface behavioural states of individuals (such as hunting, resting, socializing and travelling) in addition to acoustic recordings in order to correlate these behaviours with vocalizations.

Besides behaviour, the social composition of the group must also be taken into account [11,52]. For instance, the presence of calves and the number of animals in a group introduce variation in call structure and animals who live in sophisticated societies generally develop complex acoustic behaviours [13]. Complex vocal repertoires are known in birds [71,72], primates [73] and cetaceans. For the latter, vocal repertoires can be specific to groups even if they share a common geographical area (this is the case for killer whales [3] and sperm whales [74]), which was hypothesized to be a social identity marker serving in maintaining group integrity [75]. Long-finned pilot whales have a hierarchical social system (clans are composed of pods that are composed of matrilines [76]), which is relatively similar to that of killer whales and sperm whales. Therefore, there may exist calls specific to certain areas of the Mediterranean, corresponding to particular groups of animals, or to a change of behaviours of the same groups responding to different environments.

In addition, alike with other species, the vocal repertoire of long-finned pilot whales in the Mediterranean Sea could vary with geographic locations. Indeed, the recordings in this study were made in different areas, and figure 9 shows few calls are common between databases (8). Therefore, there may exist calls specific to certain areas of the Mediterranean, corresponding to particular groups of animals.

This hypothesis suggests a geographical variation of the repertoire, and thus the vocal repertoire of the Mediterranean population could be different from that of the Strait of Gibraltar population. It is not yet known whether repertoires are specific to clans, pods or other social units. To investigate this further, it would be imperative to conduct extensive long-term recordings across various locations in the Mediterranean Sea. This comprehensive approach would allow researchers to determine whether repertoires change over time, over locations, and if they can be attributed to specific groups (using photo-identification, for instance). The study by Baron *et al*. [53] also highlighted acoustic differences between two populations of long-finned pilot whales. These distinctions could be attributed to geographic isolation, habitat separation, or cultural drifts between neighbouring yet genetically distinct populations [77].

Photo-identification studies of long-finned pilot whales in the Mediterranean Sea are rare. To our knowledge, only one was conducted by Meglio *et al*. [78]. A total of 165 different individuals were photo-identified, of which only 13 were photographed 2 or 3 times. It is, therefore, necessary to carry out further such studies in conjunction with acoustic recordings in order to link vocalizations to clans, populations or areas.

A temporal component may also have an impact on cetaceans’ vocal repertoires, although this has not yet been demonstrated for long-finned pilot whales, but for other odontocetes. For instance, the study of Díaz López [57] showed the relationship between seasons (related to mating behaviours) and social signals in bottlenose dolphins. Moreover, Deecke *et al*. [79] have shown an inter-annual evolution of dialects in killer whales. Long-term and inter-seasonal recordings provide an interesting line of research to test this hypothesis for Mediterranean long-finned pilot whales.

The last factor to take into account is anthropogenic impacts. Human presence and noise can also influence the acoustic emissions of long-finned pilot whales (i.e. anthropic pressure on odontoceties yielding to variation of vocal pattern [80]). For example, Rendell & Gordon [55] studied the vocal response of long-finned pilot whales to military sonar in the Mediterranean Sea, and demonstrated that the number of calls varied depending on sonar emissions.

Table 3 shows this species’ vocal repertoire is fundamentally linked to its ecology and population dynamics. The acoustic study of this species can therefore provide keys to understanding the evolution of its behaviour, spatio-temporal distribution, social structure and demographic parameters. Very few studies have focused on long-finned pilot whales in the Mediterranean Sea, and characterizing their vocal repertoire is a good start to understanding how this species evolves in order to anticipate conservation measures.

### 4.5. Methodological critique and perspectives

This is the first time the method from Best *et al*. [18] has been used to discover a new call repertoire. This study shows it is effective and fast for long-finned pilot whales. The method proposed by Best *et al*. [18] is very efficient compared to manual annotation, as the pre-clusters were built in just a few minutes. Subsequently, a manual verification of the clusters was conducted, which took approximately 3 h. Furthermore, the manual method would have required several days of annotation by multiple annotators and would likely have introduced significant annotation biases [18]. This new framework has already been applied to different species in the study of Best *et al*. [18]: bengalese finch (*Lonchura striata domestica*), California thrasher (*Toxostoma redivivum*), bottlenose dolphin (*Tursiops truncatus*), humpback whales with varying numbers of calls, repertoire sizes, SNRs, and frequency ranges.

However, the method presented in this paper still has limitations. First, the opportunistic acoustic data collection makes the interpretation of repertoire size and its link with social and/or genetic structures impossible. Second, despite alleviating some effort needed to categorize all detected calls, the unsupervised clustering still requires manual intervention for the validation and correction of clusters. This is not only time-consuming, but also might induce subjective biases in the process [81]. The sparse acoustic sampling and the relatively low rate of categorization (65%) make this repertoire incomplete. Nonetheless, this first description might support the following work in the analysis of Mediterranean long-finned pilot whale calls.

Finally, with a fully passive acoustic approach, the definition of call types and their relevant parameters (e.g. considering them as frequency invariant) cannot be tested for how they are actually perceived by the animals. Monitoring of behaviour and/or individual identities would help to validate such assumptions.

This first study of long-finned pilot whale acoustics in the Mediterranean Sea opens many perspectives. The first would be to do more recordings at a larger spatio-temporal scale, with both human-operated and long-term autonomous recorders. The first allows the visual observation of animals, and, thus, to link acoustic signals to surface behaviour and groups. The latter, on the other hand, makes it possible to study the communication system’s seasonal and/or inter-annual evolution. The second perspective would be to increase the sampling frequencies of new recordings in order to study the full vocal repertoire of long-finned pilot whales (including calls and whistles above 10 kHz). Some high-frequency patterns could be explored in some parts of the data set of this study (192 kHz for Pavia, Sphyrna and WW database).

Once the vocal repertoire of this species in the Mediterranean is fully known, it will be possible to analyse sequences and, in particular, to test the hypothesis that acoustic complexity is linked to the group’s social complexity [13]. By analysing the repetition, variety and combination of calls, we could understand how communication is organized within this population, particularly when associated with surface behaviour.

This study shows the vocal repertoire of the Mediterranean long-finned pilot whale is complex and may be a good starting point to understand this species’ communication system in the area.

## Data Availability

The recordings of each type are available online at: http://sabiod.lis-lab.fr/pub/ADSIL/pilot_whales/. Each recording lasts 2 seconds, with a sampling rate of 48 000 Hz. Supplementary material is available online [82].

## Footnotes

^1^
This interface is available at https://gitlab.lis-lab.fr/paul.best/pitch_annotation/.

^2^
Some variables were subjected to a non-parametric CART using the rpart package in R [45], following a method employed in several similar studies [5,46,47].

## Dedication

We would like to dedicate this work to our friend and colleague Gianni Pavan, who helped greatly with this work, but sadly passed away before its publication.
